# Dimerized Domain V of Beta2-Glycoprotein I Is Sufficient to Upregulate Procoagulant Activity in PMA-Treated U937 Monocytes and Require Intact Residues in Two Phospholipid-Binding Loops

**DOI:** 10.3390/antib6020008

**Published:** 2017-06-02

**Authors:** Alexey Kolyada, David A. Barrios, Natalia Beglova

**Affiliations:** Department of Medicine, Beth Israel Deaconess Medical Center and Harvard Medical School, Boston, MA 02215, USA; akolyada@yahoo.com (A.K.); dabarrio@bidmc.harvard.edu (D.A.B.)

**Keywords:** beta2-glycoprotein I, antiphospholipid syndrome, antiphospholipid antibodies, anticardiolipin antibody

## Abstract

Upregulation of the procoagulant activity of monocytes by antibodies to beta2-glycoprotein I (β2GPI) is one of the mechanisms contributing to thrombosis in antiphospholipid syndrome. Current knowledge about receptors responsible for the upregulation of procoagulant activity by β2GPI/anti-β2GPI complexes and their binding sites on β2GPI is far from complete. We quantified the procoagulant activity expressed by phorbol 12-myristate 13-acetate (PMA)-differentiated U937 cells by measuring clotting kinetics in human plasma exposed to stimulated cells. Cells stimulated with anti-β2GPI were compared to cells treated with dimerized domain V of β2GPI (β2GPI-DV) or point mutants of β2GPI-DV. We demonstrated that dimerized β2GPI-DV is sufficient to induce procoagulant activity in monocytes. Using site-directed mutagenesis, we determined that the phospholipid-binding interface on β2GPI is larger than previously thought and includes Lys308 in β2GPI-DV. Intact residues in two phospholipid-binding loops of β2GPI-DV were important for the potentiation of procoagulant activity. We did not detect a correlation between the ability of β2GPI-DV variants to bind ApoER2 and potentiation of the procoagulant activity of cells. The region on β2GPI inducing procoagulant activity in monocytes can now be narrowed down to β2GPI-DV. The ability of β2GPI-DV dimers to come close to cell membrane and attach to it is important for the stimulation of procoagulant activity.

## 1. Introduction

Antiphospholipid syndrome (APS) is an autoimmune disease characterized by clinical thrombosis, recurrent fetal loss during pregnancy and the presence of antiphospholipid antibodies [1,2]. Antiphospholipid antibodies (aPL) detected by laboratory tests for APS are highly heterogeneous even in a single patient [3,4]. The majority of aPL recognize serum proteins that bind anionic phospholipids. Autoantibodies that bind directly to anionic phospholipids are often present in diseases that do not have any link to thrombosis and are generally considered irrelevant to APS [5,6,7]. Nevertheless, it was recently demonstrated that APS patients may have antibodies that bind cardiolipin without serum protein cofactor, and these antibodies are prothrombotic in mice [8]. The heterogeneity of antiphospholipid antibodies and the wide range of clinical features in APS patients suggest that there are multiple pathways leading to the disease [9,10,11].

There is a wealth of data demonstrating that anti-β2GPI antibodies are common in APS patients and that these antibodies correlate with thrombosis [12,13,14,15,16,17]. Anti-β2GPI antibodies potentiate thrombus formation in animal models of thrombosis and induce a prothrombotic state in monocytes, platelets and endothelial cells in vitro [18,19,20]. B2GPI/anti-β2GPI complexes have been reported to interact with several receptors and cell-surface molecules, such as toll-like receptors TLR2, TLR4, TLR8, ApoER2, GPIbα and annexin A2 [21]. The involvement of TLR4, ApoER2 and annexin A2 in the prothrombotic effects of anti-β2GPI antibodies is supported by in vivo studies using murine thrombosis models [22,23,24,25]. The relative contribution of these receptors to cellular activation by anti-β2GPI antibodies and the onset of thrombosis in vivo remains poorly understood. It was recently demonstrated in monocytes that endocytosis is required for anti-β2GPI signaling [26]. 

B2GPI is a serum protein consisting of five domains [27]. Flexible linkers between domains permit the β2GPI molecule to adopt different shapes. The circular shape in which domain I is adjacent to domain V is the predominant conformation of β2GPI in normal human plasma. In the pathologically-active extended conformation of β2GPI, domain V is independent of other β2GPI domains. Anti-β2GPI antibodies in APS patients with thrombosis most often bind to domain I of β2GPI [28]. Current knowledge of how β2GPI/antibody complexes interact with receptors is incomplete. It is limited to ApoER2, GPIbα and anionic phospholipids. The binding sites for these receptors were localized to domain V of β2GPI [29,30,31,32,33,34].

Induction of tissue factor (TF) in endothelial cells and monocytes is an important prothrombotic mechanism of β2GPI/anti-β2GPI complexes [35,36,37]. Monocytes isolated from APS patients have elevated expression of TF and TF-dependent procoagulant activity [38,39,40]. The ability of patients’ IgG to stimulate TF activity in monocytes in vitro correlates with the presence of clinical thrombosis and the levels of anti-β2GPI antibodies in IgG samples [41]. Experimental data implicate TLR2, TLR4, ApoER2 and annexin A2 in the upregulation of TF by anti-β2GPI antibodies [23,42,43,44]. The binding site on β2GPI for the receptor responsible for the induction of procoagulant activity is unknown. Indefinite anticoagulation, which is a treatment of choice for high risk APS patients, is not always effective in preventing the recurrence of thrombosis [45,46,47]. A detailed understanding of how β2GPI interacts with receptors involved in cellular activation by β2GPI/anti-β2GPI complexes is essential for the development of drugs specific for antiphospholipid syndrome. 

In this study, we compared dimerized β2GPI-DV to β2GPI/anti-β2GPI complexes by its ability to stimulate procoagulant activity in phorbol 12-myristate 13-acetate (PMA)-differentiated U937 cells. U937 is a monocytic cell line, where cells are arrested at an early stage of differentiation. Treatment with PMA induces differentiation of U937 cells to monocytes/macrophages characterized by expression of CD14 and CD11a, CD11b and CD18 integrins [48]. U937 monocytes express all receptors that were suggested to interact with β2GPI/anti-β2GPI complexes [49,50,51,52] and respond to antibodies isolated from APS patients with thrombosis by upregulating TF [41]. We used site-directed mutagenesis to change residues in β2GPI-DV involved in binding to ApoER2 and anionic phospholipids and compared how these mutations affected the induction of procoagulant activity expressed by PMA-differentiated U937 cells. 

## 2. Results

### 2.1. In the Presence of Dimerizing Antibodies, Domain V of β2GPI Is Sufficient to Stimulate the Procoagulant Activity of PMA-Differentiated U937 Monocytes 

**Figure 1:**
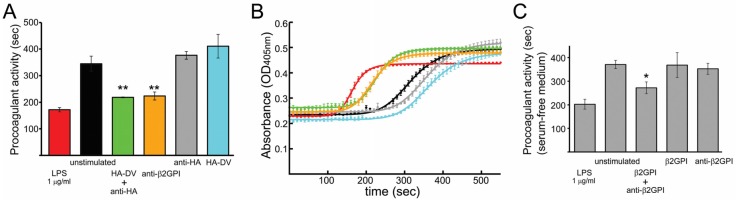
Procoagulant activity induced in U937 cells. (**A**) Cell culture medium contained 10% normal human serum, which was a source of β2GPI. PMA-treated U937 cells were incubated for 6 h with LPS (1 μg/mL); medium; HA-DV (8 μg/mL) with anti-HA (14 μg/mL); anti-β2GPI (16 μg/mL); anti-HA (14 μg/mL) alone; and HA-DV (8 μg/mL) alone. ** *p* < 0.001 compared to medium, HA-DV only and anti-HA only; (**B**) Example of coagulation kinetics curves measured in one of the three experiments used to quantify procoagulant activity in (A). From left to right are the kinetics curves corresponding to cells treated with LPS (red); HA-DV with anti-HA (green); anti-β2GPI (orange); medium (black); anti-HA alone (gray); and HA-DV alone (cyan). Each data point represents the mean and the deviation from the mean of measurements performed in duplicates; (**C**) Procoagulant activity induced by anti-β2GPI in serum-free medium depends on the presence of β2GPI. PMA-treated U937 cells were incubated for 6 h in serum-free medium supplemented with LPS (1 μg/mL); medium; β2GPI (20 μg/mL) with anti-β2GPI (16 μg/mL); β2GPI (20 μg/mL) alone; and anti-β2GPI (16 μg/mL) alone. * *p* < 0.05 compared to medium and β2GPI alone. Procoagulant activities in (A,C) were quantified from coagulation kinetics curves and expressed as time to half-maximal coagulation. Values represent mean ± SD (*n* = 3).

To mimic dimerized domain V in β2GPI/anti-β2GPI complexes, we attached an HA-tag (amino acid sequence YPYDVPDYA) at the N-terminus of domain V and used anti-HA-tag antibodies to form HA-DV dimers. The procoagulant activity induced by HA-DV/anti-HA complexes in U937 cells was compared to that induced by β2GPI/anti-β2GPI complexes (Figure 1A,B). The procoagulant activity was quantified using coagulation kinetics curves (Figure 1B). Each experimental condition was characterized by the time required to achieve half-maximal coagulation. In our preliminary studies, we performed dose response experiments to determine the concentrations of anti-β2GPI and anti-HA antibodies that are necessary to induce the same level of procoagulant activity in cells as the procoagulant activity induced by 1 μg/mL of LPS. We found the needed concentrations to be approximately 100 nM, which is what we used in our studies. The concentrations of β2GPI and HA-DV (~0.4 μM of β2GPI in 10% serum and 0.8 μM of HA-DV) were in excess of the concentrations of anti-β2GPI and anti-HA antibodies used, so that antibodies were fully loaded with the antigen.

Normal human plasma exposed to cells treated with anti-β2GPI or anti-HA-tag antibodies in the presence of HA-DV coagulated significantly faster than plasma exposed to untreated cells (Figure 1A). Treating cells with either HA-DV alone or anti-HA antibodies alone did not change coagulation time compared to untreated cells. The acceleration of coagulation by monocytes stimulated with anti-β2GPI antibodies was β2GPI dependent (Figure 1C). When cells were stimulated under serum-free conditions, only cells exposed to both β2GPI and anti-β2GPI antibodies significantly accelerated coagulation compared to untreated cells. Neither anti-β2GPI antibodies nor β2GPI alone had any effect on coagulation time in a serum-free medium. 

### 2.2. Measured Procoagulant Activity of U937 Cells Is TF-Dependent

**Figure 2:**
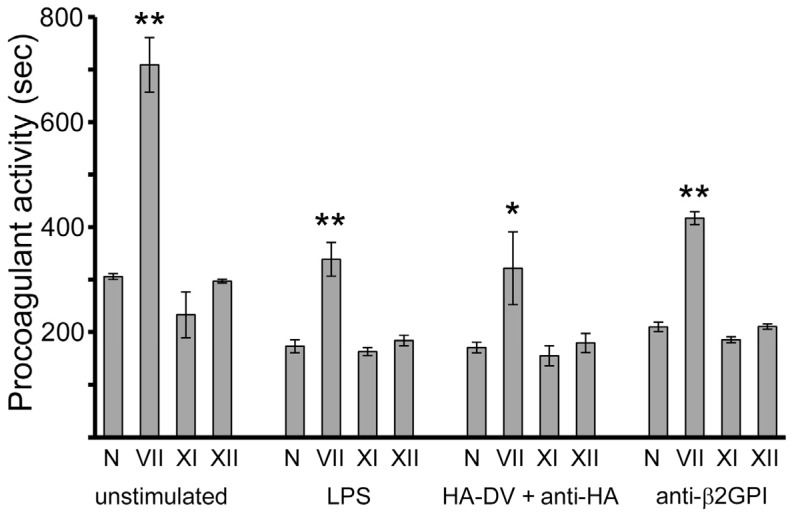
Procoagulant activity exhibited by U937 cells is caused by the upregulation of cell-surface tissue factor. Cell culture medium contained 10% normal human serum. PMA-treated U937 cells were stimulated for 6 h with medium only; LPS (1 μg/mL); HA-DV (8 μg/mL) with anti-HA (14 μg/mL); and anti-β2GPI (16 μg/mL). The procoagulant activities of the cells were measured in pooled normal human plasma (N), as well as in plasmas deficient in factors VII, XI and XII. ** *p* < 0.001, * *p* < 0.01, compared to normal plasma.

The coagulation cascade consists of two pathways leading to the formation of the fibrin clot: the intrinsic and extrinsic pathways. The intrinsic clotting pathway is activated by the contact activation of FXII, and the extrinsic clotting pathway is initiated by the TF/FVIIa complex. To differentiate the contribution of FXII-dependent and TF-dependent pathways to the initiation of the measured procoagulant activity of the treated U937 cells, we used plasmas deficient in factors FVII, FXII and FXI. Deficient plasmas were exposed to U937 cells stimulated for 6 h with test reagents. The coagulation kinetics of deficient plasmas were compared to the coagulation kinetics of normal plasma (Figure 2). For all stimulants (LPS, HA-DV/anti-HA complexes, anti-β2GPI and untreated cells), factor FVII-deficient plasma exposed to stimulated cells clotted significantly more slowly than normal plasma. The absence of factors FXI or FXII had no effect on the clotting kinetics, when compared to the clotting kinetics of normal plasma. These results demonstrated that the TF/FVIIa complex formed on the surface of U937 cells was the major activator of the clotting cascade in our experiments. Therefore, our assay detects the procoagulant activity of cell-surface TF, which is upregulated by the treatment with anti-β2GPI antibodies and with dimerized β2GPI-DV (Figure 1A).

### 2.3. TNFα Released by U937 Cells Stimulated with either HA-DV/Anti-HA Complexes or Anti-β2GPI Antibodies Was Negligible Compared to TNFα Released by Cells Stimulated with TLR4 and TLR2 Ligands

**Figure 3:**
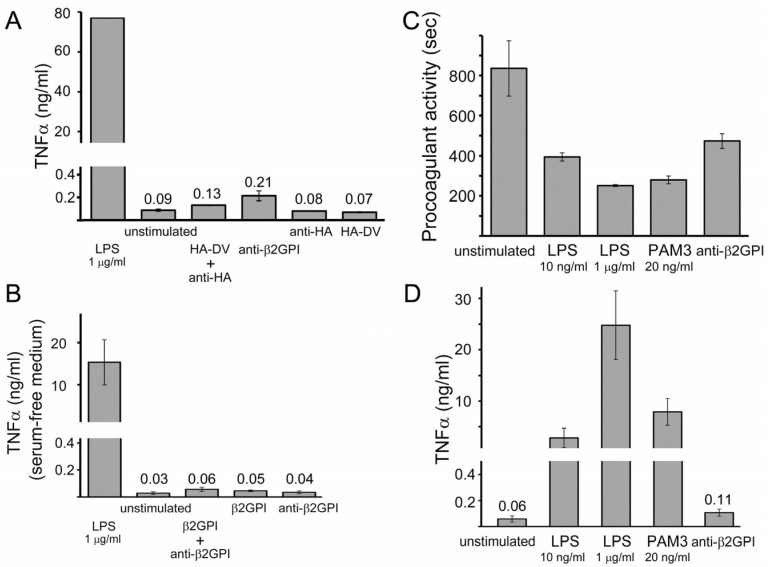
TNFα released into the medium from U937 cells. (**A**) Cell culture medium contained 10% normal human serum. PMA-treated U937 cells were incubated for 6 h with LPS (1 μg/mL); medium; HA-DV (8 μg/mL) with anti-HA (14 μg/mL); anti-β2GPI (16 μg/mL); anti-HA (14 μg/mL) alone; and HA-DV (8 μg/mL) alone; (**B**) PMA-treated U937 cells were incubated for 6 h in a serum-free medium supplemented with LPS (1 μg/mL); medium; β2GPI (20 μg/mL) with anti-β2GPI (16 μg/mL); β2GPI (20 μg/mL) alone; and anti-β2GPI (16 μg/mL) alone. Values represent mean ± SD (*n* = 3); (**C**,**D**) Procoagulant activities (C) and TNFα released into the medium (D). PMA-treated U937 cells were incubated for 6 h with medium; LPS (10 ng/mL); LPS (1 μg/mL); Pam3CSK4 (20 ng/mL); and anti-β2GPI (16 μg/mL). Results are expressed as the mean ± deviation from the mean (*n* = 2). Mean value of released TNFα is specified at the top of a column.

We measured the amount of TNFα released by cells stimulated with either anti-β2GPI antibodies or HA-DV/anti-HA complexes and compared it to the amounts of TNFα secreted by cells stimulated with the TLR4-specific ligand LPS and the TLR2-specific ligand Pam3CSK4 (Figure 3A–D). LPS and Pam3CSK4 upregulated procoagulant activity and induced a massive release of TNFα from U937 cells (Figure 3C,D). Interestingly, although the procoagulant activity induced by anti-β2GPI antibodies and by HA-DV/anti-HA complexes was similar to that induced by LPS, neither anti-β2GPI antibodies nor HA-DV/anti-HA complexes induced appreciable release of TNFα from U937 cells. 

### 2.4. Design and Characterization of Point Mutants of Domain V of β2GPI (HA-DV)

**Figure 4:**
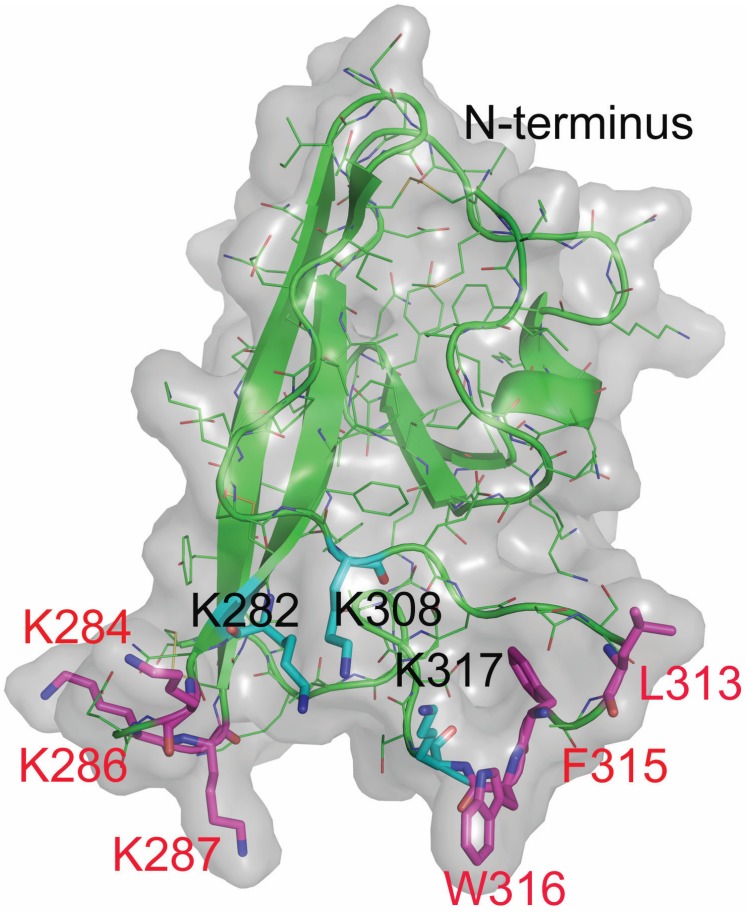
Structure of domain V of β2GPI (cartoon representation). The transparent molecular surface of domain V of β2GPI is colored gray. The residues interacting with A1 (K308, K317 and K282, colored cyan) and the residues in two phospholipid-binding loops (K284, K286, K287 and L313, F315, W316, colored magenta) are rendered as sticks.

Information, detailed at the amino acid resolution, on how β2GPI interacts with cells is limited to ApoER2 and anionic phospholipids [29,30,32,34,53]. Domain V of β2GPI contains residues critical for the binding to ApoER2 and anionic phospholipids (Figure 4). U937 cells express two isoforms of ApoER2 [52], each of which contains the β2GPI-binding module A1 in the ligand-binding domain [54]. We made point mutants of the HA-tagged domain V of β2GPI (HA-DV) with the goal of dissecting the contribution of ApoER2 and anionic phospholipids to potentiation of procoagulant activity in monocytes treated with dimerized HA-DV. The selected residues were Lys308 and Lys282, which are involved in the binding of β2GPI domain V to ApoER2 [34,53] and the residues in two phospholipid-binding loops (Figure 4). One of the phospholipid-binding loops contains basic residues Lys284, Lys286 and Lys287, and the other loop is composed of a hydrophobic sequence between the residues Leu313 and Trp316 [29,30,32].

#### 2.4.1. The Binding of HA-DV Variants to ApoER2

**Figure 5:**
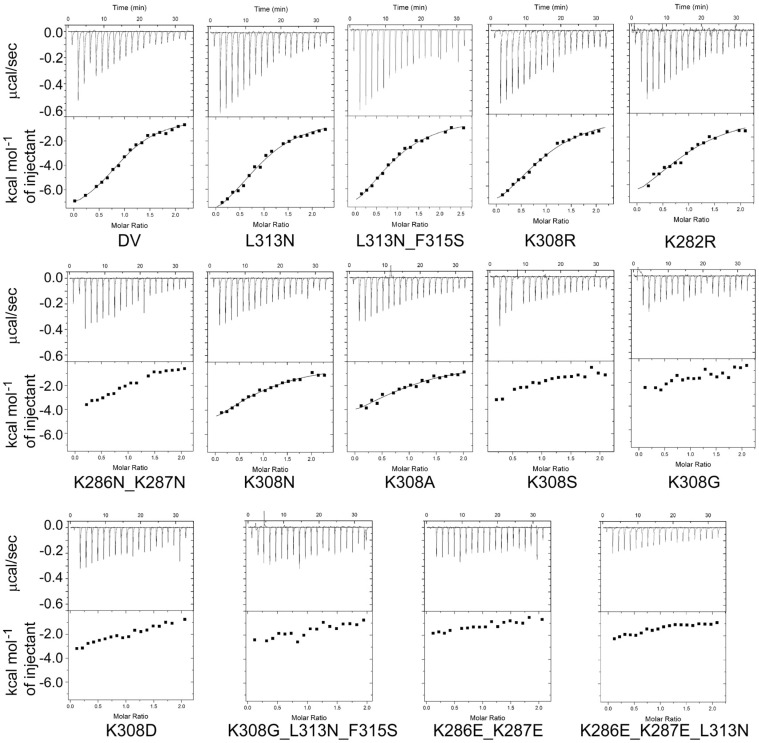
Binding of A1 to HA-DV variants. Isothermal titration calorimetry (ITC) titrations of HA-DV variants (50 μM) in a sample cell with A1 (500 μM) in the injection syringe.

A1 is a polypeptide that closely resembles the β2GPI-binding module from ApoER2 [53,54]. The ability of HA-DV variants to bind ApoER2 was evaluated by comparing their ability to bind A1. The binding affinity between HA-DV variants and A1 was evaluated by isothermal titration calorimetry (ITC) (Figure 5). ITC is used to directly measure the heat released or absorbed, when binding occurs. HA-DV or HA-DV variants were placed in a sample cell and titrated with A1. Heat changes were detected and measured. First, we measured the binding curve and calculated the binding constant for the HA-DV/A1 complex. We then used the same experimental conditions to compare HA-DV variants to HA-DV with respect to their ability to bind A1. The quantity of heat released upon binding, which is measured by ITC, is directly proportional to the amount of binding. We compared titration curves measured for HA-DV mutants to the titration curve measured for HA-DV. Four mutants of HA-DV bound A1 with affinity similar to that of wild type HA-DV. These were the two HA-DV variants with conservative Lys to Arg mutations (Lys308/Arg and Lys282/Arg) and the two HA-DV variants with mutations in one of the two phospholipid-binding loops (Leu313/Asn and Leu313/Asp_Phe315/Ser). The shallow slope of titration curves measured for all other studied mutations in HA-DV strongly suggests that these mutations disrupted the binding of HA-DV mutants to A1. These results confirmed our previous observations that the hydrophobic loop Leu313-Phe315 is far from the binding interface in the HA-DV/A1 complex [34,53]. 

#### 2.4.2. The Binding of HA-DV Variants to Cardiolipin

**Figure 6:**
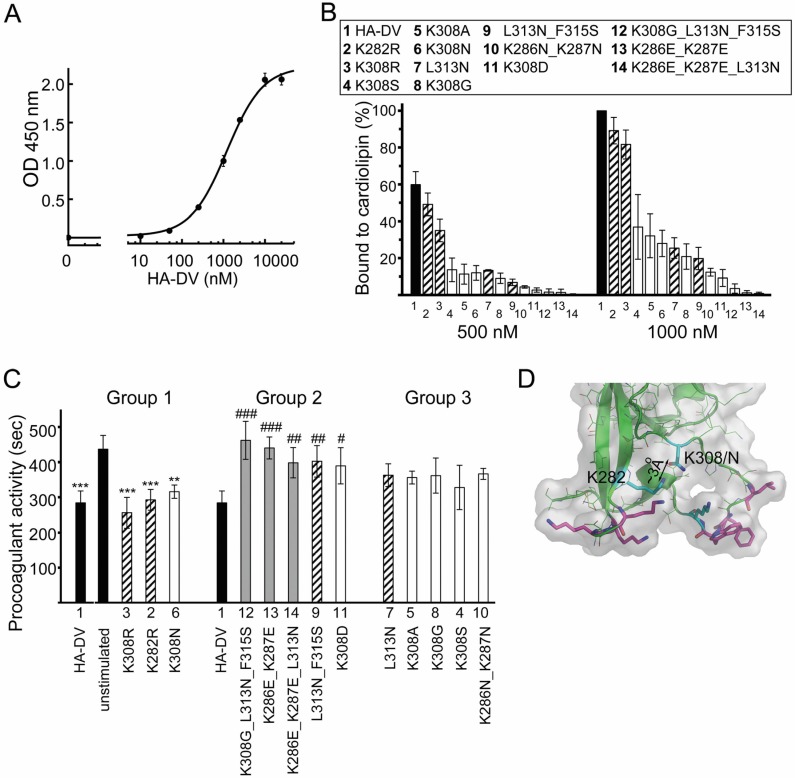
The ability of HA-DV variants to bind cardiolipin and induce procoagulant activity in U937 cells. (**A**) The binding of wild type HA-DV to cardiolipin. The half-maximal binding was achieved at 1.2 μM of HA-DV. Each data point shows the mean ± deviation from the mean of two measurements; (**B**) The ability of HA-DV variants to bind cardiolipin compared to wild type HA-DV (black columns). HA-DV variants that bind A1 are designated by hatched columns. Results are expressed as the percentage of cardiolipin binding measured for wild type HA-DV at 1000 nM. Values represent the mean ± SD (*n* = 3). HA-DV mutants are numbered as in (**C**); (**C**) Procoagulant activity in cells treated with HA-DV variants (8 μg/mL) in the presence of anti-HA (14 μg/mL). *** *p* < 0.001 and ** *p* < 0.01 compared to medium. ^###^ *p* < 0.001, ^##^ *p* < 0.01 and ^#^ *p* < 0.05 compared to wild type HA-DV in the presence of anti-HA. HA-DV variants are numbered as in (B). HA-DV variants that bind A1 (hatched columns). HA-DV variants that have less than 4% of cardiolipin binding (gray columns). Values represent the mean ± SD (*n* = 3); (**D**) Putative hydrogen bond formed between the sidechains of residues K282 and N308 in the K308/N mutant of HA-DV. The transparent molecular surface of domain V of β2GPI is colored gray. Sidechains interacting with A1 (cyan) and with phospholipids (magenta) are rendered as sticks.

Next, we analyzed the ability of HA-DV mutants to bind the anionic phospholipid cardiolipin compared to wild type HA-DV. Half-maximal binding to cardiolipin was achieved at a 1.2 μM concentration of wild type HA-DV (Figure 6A). From the cardiolipin-binding curve measured for wild type HA-DV, we selected two concentrations, 500 nM and 1000 nM, which fall in the linear region of the binding curve. The cardiolipin binding ability of HA-DV variants was compared to HA-DV at protein concentrations of 500 nM and 1000 nM (Figure 6B). Only the Lys308/Arg and Lys282/Arg variants of HA-DV retained cardiolipin binding activity similar to that of wild type HA-DV (Figure 6B). Any other residue besides Arg in place of Lys308 dramatically reduced the cardiolipin binding of mutated HA-DV, strongly suggesting that Lys308 is part of the phospholipid-binding interface on β2GPI. Mutations in either of the two phospholipid-binding loops disrupted the binding of HA-DV mutants to cardiolipin, as expected. Three mutants (Lys286/Glu_Lys287/Glu, Lys286/Glu_Lys287/Glu_Leu313/Asn and Lys308/Gly_Leu313/Asn_Phe315/Ser) retained less than 4% of cardiolipin-binding activity compared to wild type HA-DV.

### 2.5. ApoER2 Does Not Contribute to Upregulation of Procoagulant Activity in U937 Cells

We evaluated how point mutations in domain V affected the ability of HA-DV dimers to stimulate procoagulant activity in U937 cells. As illustrated by Figure 6C, HA-DV variants can be divided into three groups based on their ability to induce procoagulant activity in cells stimulated in the presence of dimerizing anti-HA antibodies. These are (Group 1) HA-DV variants that stimulated cells like wild type HA-DV (the difference in procoagulant activity between unstimulated cells and cells stimulated with HA-DV variants was statistically significant), (Group 2) HA-DV variants that induced procoagulant activity similar to that exhibited by unstimulated cells (the difference in procoagulant activity induced in cells stimulated with wild type HA-DV and cells stimulated with HA-DV variants was statistically significant) and (Group 3) intermediate HA-DV variants, whose activity in cells was not statistically different from either untreated cells or cells stimulated with wild type HA-DV. In the absence of dimerizing anti-HA antibodies, neither of the HA-DV mutants induced procoagulant activity statistically different from procoagulant activity exhibited by unstimulated cells, as we have already demonstrated previously.

HA-DV variants that retained their ability to bind A1 and, therefore, were capable of interacting with ApoER2 (hatched columns on Figure 6C) were distributed among all three groups of HA-DV variants. This result suggests that the binding of HA-DV/anti-HA complexes to ApoER2 is not important for the induction of procoagulant activity in U937 cells. 

### 2.6. Intact Residues in the Two Phospholipid-Binding Loops of HA-DV Are Important for the Ability of HA-DV/Anti-HA Complexes to Induce Procoagulant Activity in U937 Cells

The pathological function of β2GPI is a result of both dimerization of two β2GPI molecules by antibodies and functional interactions with receptors and phospholipids. In our system, the binding of antibodies to the epitope tag creates HA-DV/anti-HA complexes in solution, allowing us to focus on the functional interactions. 

It is clear from Figure 6C that mutation in either of the two phospholipid-binding loops in HA-DV resulted in a dimer that does not upregulate procoagulant activity of U937 cells. The ability of mutants to come close to the cell membrane and bind to it, at least to some extent, is important for stimulating the procoagulant activity. Three out of the five mutants that failed to stimulate procoagulant activity (Group 2, Figure 6C) have charge reversal mutations. All five mutants retained less than 20% of the cardiolipin-binding ability of wild type HA-DV, and three of these mutants (gray columns on Figure 6C) retained less than 4% of the cardiolipin binding. 

Three of the studied mutants, Lys308/Arg, Lys282/Arg and Lys308/Asn, were as good as wild type HA-DV in stimulating the procoagulant activity in U937 cells (Group 1, Figure 6C). A conservative Lys to Arg mutation often has little effect on protein function explaining why Lys308/Arg and Lys282/Arg mutants closely resemble wild type HA-DV in stimulating procoagulant activity. On the other hand, the Lys308/Asn mutant showed significantly reduced ability to bind cardiolipin, but retained its ability to stimulate procoagulant activity in monocytes. We found an explanation for this result by analyzing the structure of domain V of β2GPI available in the PDB data bank (PDB ID 1C1Z, 1QUB, 3OP8 and 2KRI). The Lys308/Asn mutant, compared to other less potent Lys308 mutants (Lys308/Ala, Lys308/Gly and Lys308/Ser), is capable of forming a hydrogen bond with the sidechain of Lys282. In the Lys308/Asn mutant, this hydrogen bond combined with phospholipid-bound Leu313 restricts flexibility in the unstructured region between the residues 308 and 313 (Figure 6D). This unstructured region is stabilized by the binding of Lys308 and Leu313 to anionic phospholipids in wild type HA-DV. It is likely that the region between residues 308 and 313 is in the vicinity of the binding site for the receptor, because its flexibility affects the ability of HA-DV dimers to stimulate procoagulant activity in treated cells.

## 3. Discussion

We demonstrated that domain V of β2GPI (β2GPI-DV) dimerized to mimic domain V in β2GPI/anti-β2GPI complexes is sufficient to induce procoagulant activity in PMA-differentiated U937 monocytic cells. Our data considerably simplify the search for the residues on β2GPI, which are involved in the upregulation of procoagulant activity by anti-β2GPI antibodies. The use of β2GPI-DV dimers instead of a full-length β2GPI can also simplify the search for receptors involved in the upregulation of procoagulant activity in monocytes by anti-β2GPI antibodies. This is a step towards understanding how β2GPI/anti-β2GPI complexes interact with receptors and, ultimately, towards a drug to treat anti-β2GPI-related thrombosis in APS. 

Using site-directed mutagenesis, we changed individual residues in β2GPI-DV involved in the binding of β2GPI to ApoER2 and anionic phospholipids and compared the procoagulant activity induced by dimerized β2GPI-DV variants in treated U937 cells. It has been previously shown that domain V of β2GPI is important for stimulating platelet adhesion to collagen by dimeric β2GPI and that the increase of platelet adhesion is mediated by ApoER2 [33,55]. We did not find a correlation between the ability of a β2GPI-DV variant to stimulate procoagulant activity of monocytic cells and its ability to bind A1, which is the β2GPI-binding module from ApoER2. Our results suggest that binding to ApoER2 is not important for stimulating the procoagulant activity of monocytic cells by anti-β2GPI antibodies, highlighting the complexity of molecular mechanisms of thrombosis in antiphospholipid syndrome.

The surface area on β2GPI-DV involved in phospholipid binding is much larger than previously thought. Our data suggest that Lys308 actively participates in binding of β2GPI-DV to anionic phospholipids. When Lys308 was mutated to either Ser, Ala, Asn, Gly or Asp, cardiolipin binding was reduced to 37%, 32%, 28%, 21% and 9% of the level of wild type β2GPI-DV, respectively. 

Our results show that the ability of mutants to come close to the cell membrane and attach to it, even moderately, is important for the stimulation of procoagulant activity. All active mutants in Group 1 in Figure 6C retained native residues in both phospholipid-binding loops, compared to inactive mutants in Group 2. 

It has been previously shown that the binding of β2GPI to anionic phospholipids has two effects: (1) it causes conformational rearrangement of a full-length β2GPI to expose an epitope for anti-β2GPI antibodies otherwise hidden on β2GPI [56] and (2) creates local density of β2GPI to facilitate the formation of multivalent complexes with low affinity APS antibodies [57,58,59]. Using antibodies to an unobstructed HA-tag attached to β2GPI-DV, we have shown that the ability of β2GPI-DV to reach the cell membrane and attach to it is important for signaling by β2GPI/anti-β2GPI complexes. 

Our data suggest that the binding of β2GPI-DV to anionic phospholipids restricts the flexibility of the unstructured region in β2GPI-DV between the residues 308 and 313 influencing the ability of dimerized domain V of β2GPI to stimulate procoagulant activity in U937 cells. Stabilization of this region is achieved in wild type β2GPI-DV by anchoring Lys308 and Leu313 to a phospholipid membrane. In the Lys308/Asn mutant, which stimulates the procoagulant activity of cells similar to wild type β2GPI-DV, the unstructured region between the residues 308 and 313 is stabilized by a hydrogen bond constraining the Asn308 residue and by phospholipid-bound Leu313. Since the flexibility of the stretch of residues between Lys308 and Leu313 influences the ability β2GPI-DV dimers to stimulate procoagulant activity, this region in β2GPI-DV is likely close to the binding site for a cell-surface receptor. 

**Figure 7:**
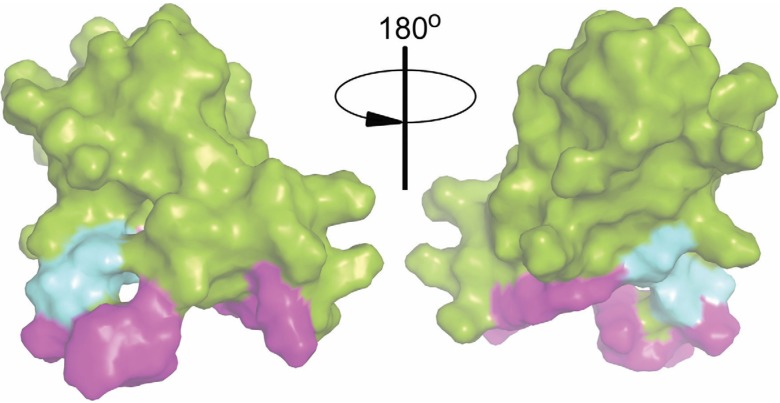
Surface representation of β2GPI-DV. The surface area around residues involved in binding to anionic phospholipids is colored magenta; the unstructured region between Lys308 and Leu313 is colored cyan.

Our results suggest a model in which β2GPI binds by its domain V to anionic phospholipids on cellular surfaces, most likely to lipid rafts enriched in anionic phospholipids and signaling proteins [60]. The binding to anionic phospholipids restricts flexibility in the unstructured loop between phospholipid-bound residues Lys308 and Leu313 in β2GPI-DV, predisposing β2GPI-DV for binding to a receptor. Anti-β2GPI antibodies keep β2GPI attached to cellular membranes by increasing the avidity of β2GPI/antibody complexes for anionic phospholipids. Binding to a receptor occurs very close to the cellular surface, because β2GPI-DV has to be attached to anionic phospholipids in order to interact with a receptor (Figure 7). Whether the interaction of β2GPI-DV with cell-surface receptors leads directly to the stimulation of procoagulant activity in cells or facilitates the endocytosis of dimerized β2GPI-DV, which then signals from endosomes, awaits further investigation.

The procoagulant activity induced in monocytes by anti-β2GPI antibodies depends on cell-surface TF. In isolated normal peripheral blood mononuclear cells (PBMC), anti-β2GPI antibodies significantly increased cell-surface TF activity and TF mRNA levels [42,61,62]. We have shown that cell-surface TF is a major contributor to the increased procoagulant activity of PMA-differentiated U937 monocytic cells treated with anti-β2GPI antibodies and β2GPI-DV dimers. The mechanism by which treatment with anti-β2GPI antibodies and β2GPI-DV dimers affects TF in U937 cells is not yet clear. We will continue investigating the extent to which treatment with anti-β2GPI antibodies contributes to de novo synthesis of TF versus decryption of TF already present on the cellular surface. Activation of cell-surface TF by anti-β2GPI antibodies and β2GPI-DV dimers could be accompanied by an increase in surface exposure of anionic phospholipids additionally contributing to procoagulant activity in the treated cells. 

It is not yet clear what receptor in monocytes is responsible for the induction of procoagulant activity by anti-β2GPI antibodies and β2GPI-DV dimers. Experiments in PBMC implicate TLR2, TLR4 and TLR8 in the upregulation of TF by anti-β2GPI antibodies, which is accompanied by a TNFα release ranging from 0.4–10 ng/mL [26,42,44,63]. TLR8 is a likely endosomal receptor for β2GPI/anti-β2GPI complexes [63]. It is also possible that endosomes have a not yet identified receptor contributing to monocyte activation by anti-β2GPI antibodies. We found that β2GPI/anti-β2GPI complexes, dimerized β2GPI-DV, LPS and Pam3CSK4 all induced procoagulant activity in PMA-differentiated U937 cells. However, β2GPI/anti-β2GPI complexes and dimerized β2GPI-DV did not promote the release of TNFα, in contrast to LPS and Pam3CSK4, which caused a massive release of TNFα into the cell culture medium. Our results suggest that another receptor, besides TLR4 and TLR2, can contribute to the upregulation of procoagulant activity in monocytes by β2GPI/antibody complexes and that the stimulation of this receptor does not lead to NF-κB activation. More investigation is required into the details of the signaling pathways induced by β2GPI/anti-β2GPI complexes and by dimers of β2GPI-DV in PMA-differentiated U937 cells and how they compare to the signaling pathways induced in PBMC. 

In conclusion, our studies in PMA-differentiated U937 monocytes have narrowed the location of the region on β2GPI responsible for the induction of procoagulant activity in monocytes by β2GPI/anti-β2GPI complexes down to domain V. Intact residues in β2GPI-DV that bind to anionic phospholipids are important for the potentiation of procoagulant activity in monocytes. The binding site for a cell-surface receptor on β2GPI-DV is likely located in the vicinity of an unstructured region in β2GPI-DV between residues 308 and 313. The flexibility of this region, which is restricted in phospholipid-bound β2GPI-DV, affects the ability of dimerized β2GPI-DV to stimulate procoagulant activity on monocytes. Our data suggest that ApoER2 is not important for the potentiation of procoagulant activity in PMA-differentiated U937 cells. The identity of the receptor that plays a role in stimulating procoagulant activity in U937 cells and the signaling pathways initiated by β2GPI/anti-β2GPI complexes and β2GPI-DV dimers awaits further investigation.

## 4. Materials and Methods 

### 4.1. Proteins

A1 is a fragment of mouse ApoER2 (residues 12–47) in which Asp is substituted for Asn36. A1 was expressed in *Escherichia coli* and purified as previously described [64]. HA-DV consists of an HA tag (amino acid sequence YPYDVPDYA) added to the N-terminus of domain V of human β2GPI (residues 244–326). HA-DV was subcloned into a pET15b vector (Novagen) in which the sequence recognized by the tobacco etch virus (TEV) protease was added after an N-terminal histidine tag so that the tag can be removed. The HA-DV protein and point mutants of HA-DV were expressed and purified as previously described [34]. 

### 4.2. Cells and Culture Conditions

The immortalized human monocyte U937 cells (ATCC, Manassas, VA, USA) were cultured in RPMI 1640 medium supplemented with 10% fetal bovine serum (FBS) (Atlanta Biologicals, Flowery Branch, GA, USA), penicillin-streptomycin and l-glutamine (Gibco, ThermoFisher Scientific, Waltham, MA, USA) at 37 °C in a humidified atmosphere with 5% CO_2_. Cells were seeded at a concentration of 5 × 10^5^ mL^−1^ and treated for 72 h with 100 nM phorbol 12-myristate 13-acetate (PMA) (Enzo Life Sciences, Farmingdale, NY, USA). After 72 h, nonadherent cells were removed along with the medium. A fresh medium containing 10% FBS was added to the cells, and adherent cells were detached by gentle pipetting. Cells were pelleted and resuspended in RPMI medium. Differentiated U937 monocytes at a concentration of 1 × 10^6^ mL^−1^ were incubated for 6 h at 37 °C in a humidified atmosphere with 5% CO_2_ in RPMI medium supplemented with 10% pooled normal human serum (Innovative Research, Novi, MI, USA) and test reagents as indicated. Human serum in cell culture media supplied β2GPI. When specified, cells were incubated in a serum free medium with or without purified β2GPI (Haematologic Technologies, Essex Junction, VT, USA), exchanged into a 20 mM Hepes, 150 mM NaCl, pH 7.5 buffer using a Zeba spin desalting column (ThermoFisher Scientific, Waltham, MA, USA) and added to the assay at a final concentration of 20 μg/mL. 

HA-DV and HA-DV mutants were used at an 8 μg/mL concentration measured by NanoDrop (ThermoScientific, Wilmington, DE, USA). The TLR2-specific ligand Pam3CSK4 was from InvivoGen, San Diego, CA, USA. Anti-HA antibody (Bethyl Laboratories, Montgomery, TX, USA) was exchanged into a 20 mM Hepes, 150 mM NaCl, pH 7.5 buffer using a Zeba spin desalting column to remove sodium azide. Goat anti-β2GPI (CL2001AP, Cedarlane Laboratories, Burlington, NC, USA) was raised against human β2GPI and affinity purified on immobilized β2GPI. LPS from *Salmonella enterica* (Sigma, St. Louis, MO, USA) was used as a positive control. Endotoxin levels in test reagents were measured with the Limulus Amebocyte Lysate (LAL) chromogenic endotoxin quantification kit (ThermoFisher Scientific, Waltham, MA, USA) at concentrations used in the assays. Endotoxin levels in HA-DV and all HA-DV mutants were below the detection limit of 0.1 EUmL^−1^, except for Lys286/Glu-Lys287/Glu and Lys286/Asn-Lys287/Asn, for which measured endotoxin was 0.5 EUmL^−1^. Endotoxin levels in β2GPI, anti-β2GPI and anti-HA were 0.15, 0.25 and 0.6 EUmL^−1^, respectively. Endotoxin in test reagents was far below 1.5 EUmL^−1^, which corresponds to 1 ng/mL of LPS from *Salmonella enterica*. This amount of LPS did not have a statistically significant effect on U937 cells.

### 4.3. Measurements of the Procoagulant Activity of U937 Cells 

After incubating for 6 h with test reagents, cells were pelleted, washed with RPMI and counted, and their viability, which was at least 90% in reported experiments, was assessed by Trypan Blue (ThermoFisher Scientific, Waltham, MA, USA). The procoagulant activity expressed by cells was quantified by measuring clotting kinetics in pooled normal platelet-poor human plasma anticoagulated with sodium citrate (Innovative Research, Novi, MI, USA). When specified, plasma depleted of factors VII, XI or XII (Haematologic Technologies, Essex Junction, VT, USA) was used in clotting studies. Clotting kinetics were measured at 37 °C using 96-well ELISA plates and a Spectramax 340PC Microplate Reader (Molecular Devices Inc., Sunnyvale, CA, USA). Human plasma, 50 μL, was added to 50 μL of cells (2 × 10^6^ mL^−1^) suspended in serum-free RPMI. The mixture was incubated for 3 min at 37 °C, and coagulation was initiated by adding 50 μL of 40 mM CaCl_2_ in 20 mM Hepes, 150 mM NaCl buffer, pH 7.5. Clotting kinetics were recorded by measuring absorbance at 405 nm. Kinetics data were fitted to a 4-parameter equation using the Gnuplot 5.0 program (http://www.gnuplot.info/). The time needed to achieve a half-maximal increase in OD was calculated for each kinetics curve and used to characterize the procoagulant activity of the cells.

### 4.4. TNFα ELISA

After 6 h of stimulation with test reagents, cells were pelleted, and the supernatant was collected and stored frozen at −80 °C until use. The concentration of TNFα released into media was quantified with Quantikine ELISA (R&D Systems, Minneapolis, MN, USA). 

### 4.5. Isothermal Titration Calorimetry 

To measure the binding between A1 and the HA-tagged domain V of β2GPI (HA-DV) and its variants, lyophilized proteins were resuspended in a 25 mM Hepes, pH 7.1 buffer containing 50 mM NaCl and 2 mM CaCl_2_ and dialyzed overnight at 4 °C in the same buffer. Measurements were performed at 298 K using a MicroCal iTC200 system (Malvern, Malvern Instruments, U.K.). A1 at a concentration of 500 μM was placed into an injection syringe and titrated in 2 μL increments into a sample cell containing 50 μM of HA-DV or HA-DV variants. Binding isotherms were fit to a one site binding model using the Origin software for ITC. 

### 4.6. Cardiolipin ELISA

ELISA 96-well plates (Costar, Corning, NY, USA) were coated with 50 μL per well of cardiolipin (Sigma, St. Louis, MO, USA) prepared at 200 μg/mL in ethanol and blocked for 2 h with 4% BSA in a 20 mM Tris, 100 mM NaCl buffer at pH 7.4. To generate a binding curve, increasing concentrations of HA-DV were applied to wells. The binding data were fit to a one-site model using the Gnuplot 5.0 program (http://www.gnuplot.info/). Cardiolipin binding by HA-DV variants was compared to cardiolipin binding by HA-DV at protein concentrations of 500 nM and 1000 nM. Bound HA-tagged proteins were detected with HRP-conjugated anti-HA-tag antibody (ab1265, Abcam, Cambridge, MA, USA) using a TMB (3,3′,5,5′-tetramethylbenzidine) substrate. Absorbances at 450 nm were measured on a Spectramax 340PC Microplate Reader (Molecular Devices Inc., Sunnyvale, CA, USA). 

### 4.7. Statistical Analysis 

Results are presented as the mean ± standard deviation calculated from at least three independent experiments. Statistical significance was evaluated with STATA statistical software (College Station, TX, USA) using one-way ANOVA with Bonferroni correction for multiple comparisons.
